# Supplementary material for: Splice-Junction-Based Mapping of Alternative Isoforms in the Human Proteome
Source: Cell Rep. Author manuscript; Available in PMC 2020 Jan 15. (PMC6961840; doi:10.1016/j.celrep.2019.11.026)
Supplement: 2 [file NIHMS1546469-supplement-2.zip › DF1/PXD010154/Liver/Liver_62_FTCD_EPGLPVRPPNFSEGKNQEVIDAISGAITQTPGCVLLDVDAGPSTNR.pdf]

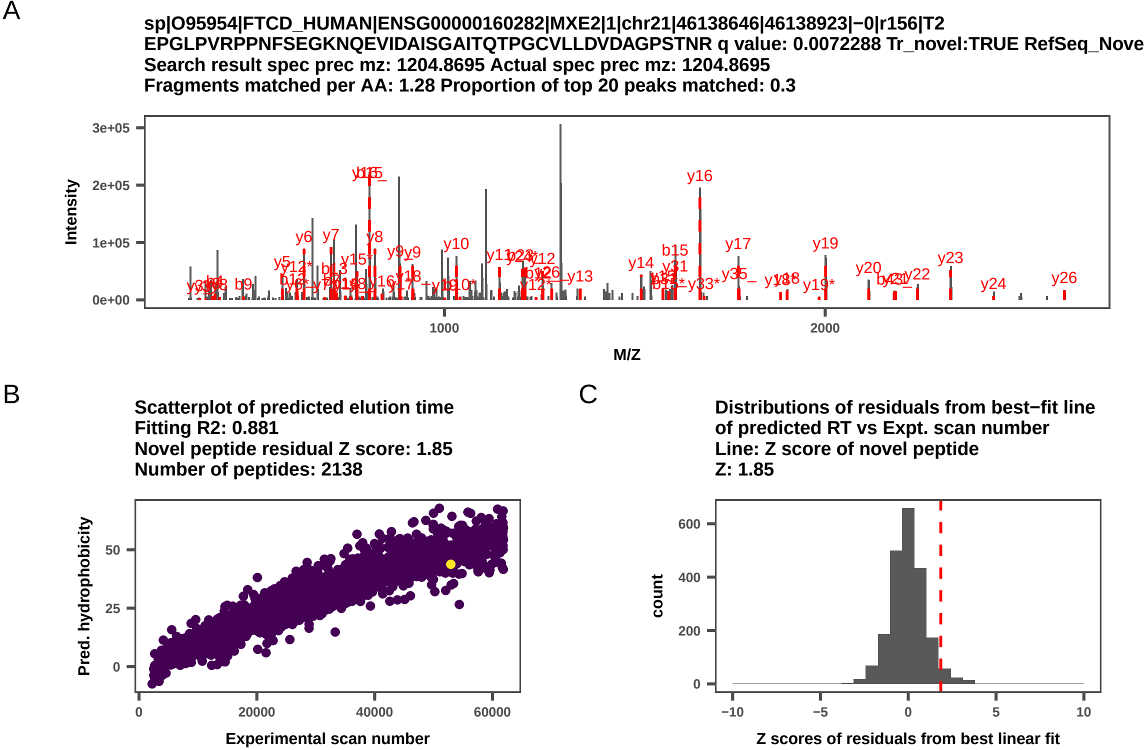
A
sp|O95954|FTCD_HUMAN|ENSG00000160282|MXE2|1|chr21|46138646|46138923|−0|r156|T2
EPGLPVRPPNFSEGKNQEVIDAISGAITQTPGCVLLDVDAGPSTNR q value: 0.0072288 Tr_novel:TRUE RefSeq_Nove
Search result spec prec mz: 1204.8695 Actual spec prec mz: 1204.8695
Fragments matched per AA: 1.28 Proportion of top 20 peaks matched: 0.3
Intensity
3e+05
2e+05
1e+05
0e+00
M/Z
1000
2000
y16
y6
y7
y8
y5
y9
y10
y12
y13
y14
b15
y31
y17
y19
y20
y22
y23
y24
y26
b9
y19*
y33*
B
Scatterplot of predicted elution time
Fitting R2: 0.881
Novel peptide residual Z score: 1.85
Number of peptides: 2138
Pred. hydrophobicity
50
25
0
Experimental scan number
0
20000
40000
60000
C
Distributions of residuals from best−fit line
of predicted RT vs Expt. scan number
Line: Z score of novel peptide
Z: 1.85
count
600
400
200
0
Z scores of residuals from best linear fit
−10
−5
0
5
10
